# Sodium Taurocholate Stimulates *Campylobacter jejuni* Outer Membrane Vesicle Production via Down-Regulation of the Maintenance of Lipid Asymmetry Pathway

**DOI:** 10.3389/fcimb.2019.00177

**Published:** 2019-05-29

**Authors:** Cadi Davies, Aidan J. Taylor, Abdi Elmi, Jody Winter, Janie Liaw, Anna D. Grabowska, Ozan Gundogdu, Brendan W. Wren, David J. Kelly, Nick Dorrell

**Affiliations:** ^1^Faculty of Infectious and Tropical Diseases, London School of Hygiene and Tropical Medicine, London, United Kingdom; ^2^Department of Molecular Biology and Biotechnology, University of Sheffield, Sheffield, United Kingdom; ^3^School of Science and Technology, Nottingham Trent University, Nottingham, United Kingdom

**Keywords:** *Campylobacter*, bile salts, outer membrane vesicles, maintenance of lipid asymmetry pathway, MlaA

## Abstract

*Campylobacter jejuni* outer membrane vesicles (OMVs) contain numerous virulence-associated proteins including the cytolethal distending toxin and three serine proteases. As *C. jejuni* lacks the classical virulence-associated secretion systems of other enteric pathogens that deliver effectors directly into target cells, OMVs may have a particularly important role in virulence. *C. jejuni* OMV production is stimulated by the presence of physiological concentrations of the bile salt sodium taurocholate (ST) through an unknown mechanism. The maintenance of lipid asymmetry (MLA) pathway has been implicated in a novel mechanism for OMV biogenesis, open to regulation by host signals. In this study we investigated the role of the MLA pathway in *C. jejuni* OMV biogenesis with ST as a potential regulator. OMV production was quantified by analyzing protein and lipid concentrations of OMV preparations and OMV particle counts produced by nanoparticle tracking analysis. Mutation of *mlaA* which encodes the outer membrane component of the MLA pathway significantly increased OMV production compared to the wild-type strain. Detergent sensitivity and membrane permeability assays confirmed the increased OMV production was not due to changes in membrane stability. The presence of 0.2% (w/v) ST increased wild-type OMV production and reduced OMV size, but did not further stimulate *mlaA* mutant OMV production or significantly alter *mlaA* mutant OMV size. qRT-PCR analysis demonstrated that the presence of ST decreased expression of both *mlaA* and *mlaC* in *C. jejuni* wild-type strains 11168 and 488. Collectively the data in this study suggests *C. jejuni* can regulate OMV production in response to host gut signals through changes in expression of the MLA pathway. As the gut bile composition is dependent on both diet and the microbiota, this study highlights the potential importance of diet and lifestyle factors on the varying disease presentations associated with gut pathogen infection.

## Introduction

*Campylobacter jejuni* is a microaerophilic Gram-negative bacterium that is the leading cause of foodborne bacterial gastroenteritis worldwide (Silva et al., 2011; Kaakoush et al., 2015). Whilst *C. jejuni* has a number of potential virulence factors, including a cytolethal distending toxin (Lindmark et al., 2009; Elmi et al., 2012), and multiple proteases (Karlyshev et al., 2014; Elmi et al., 2016, 2018), *Campylobacter jejuni* lacks the classical virulence associated secretion systems of other enteric pathogens that deliver effectors directly to target cells (Parkhill et al., 2000). A Type VI secretion system (T6SS) has been identified in a proportion of strains, however, the function and variability of T6SSs among *C. jejuni* strains is still poorly understood (Bleumink-Pluym et al., 2013; Ugarte-Ruiz et al., 2015). An alternative machinery to deliver potential virulence determinants are outer membrane vesicles (OMVs), which can act as a general secretion pathway among Gram-negative bacteria and maybe are of particular importance for *C. jejuni* virulence and survival.

OMVs are small spherical membrane-bound structures ranging in size from 10 to 500 nm in diameter formed from the outer membrane of Gram-negative bacteria and released into the extracellular environment (Kuehn and Kesty, 2005; Schwechheimer and Kuehn, 2015). OMVs provide a mechanism to deliver cargo in concentrated, selectively packaged parcels, protected from the extracellular environment with the potential for site specific targeting to receptors on cells (Kuehn and Kesty, 2005; Bomberger et al., 2009; Bonnington and Kuehn, 2014; Bitto et al., 2017). OMV production appears evolutionarily conserved in Gram-negative bacteria despite being metabolically energy consuming, suggesting OMVs have vital roles (Kulp and Kuehn, 2010; Roier et al., 2016). OMV production has been observed under a range of conditions in both pathogenic and non-pathogenic Gram-negative bacteria (Mashburn-Warren et al., 2008; Elmi et al., 2012; Altindis et al., 2014; Zakharzhevskaya et al., 2017); both on solid and in liquid media (Schooling and Beveridge, 2006; Schwechheimer and Kuehn, 2015), and in the presence or absence of stress (Schwechheimer and Kuehn, 2015). Conditions or gene mutations resulting in the absence of OMVs have not been identified. OMVs have been suggested to have a variety of functions important in survival and virulence via processes such as competition for growth (Manning and Kuehn, 2011; Kulkarni et al., 2015), immunomodulation (Koeppen et al., 2016; Tsatsaronis et al., 2018), biofilm formation (Schooling and Beveridge, 2006), bacterial communication (Mashburn and Whiteley, 2005; Mashburn-Warren et al., 2008), and the delivery of biomolecules such as toxins (Bielaszewska et al., 2017; Elmi et al., 2018).

Until recently there has been no general regulatory model for OMV biogenesis that could be widely applicable to Gram-negative bacteria. The three main OMV biogenesis models proposed previously have been based on either changes in cell wall to outer membrane (OM) linkages, stress or a species-specific mechanism. One previously proposed model suggests that the build-up of cellular components (such as misfolded proteins due to heat shock, or peptidoglycan fragments due to cell wall remodeling defects) at the OM result in bulging and subsequent blebbing (Zhou et al., 1998; Mcbroom and Kuehn, 2007). OMVs have since been demonstrated to contain biologically active cargo and not just cell waste (Mashburn and Whiteley, 2005; Bielaszewska et al., 2017; Elmi et al., 2018). A second mechanism proposed that the reduction in cell wall to OM interactions can cause areas prone to blebbing. This has been demonstrated by OMVs being released at cell division sites with high frequency (Kulp and Kuehn, 2010), however, OMV release is not restricted to cell division sites (Elhenawy et al., 2016). Deatherage et al. (2009) studied the effect of removing cell wall to OM linkages on OMV production and found removal of OmpA linkages did not compromise the cell membrane. However, removal of other linkages *(lpp, tolA, tolB*, and *pal*) did cause membrane defects. Additional studies have found that the down-regulation of *ompA* is dependent on the membrane stress sigma factor, σ^E^ (Song et al., 2008). A further mechanism has been identified in the OM of *Pseudomonas aeruginosa*, which has increased fluidity compared to the OM of other Gram-negative bacteria. *Pseudomonas aeruginosa* produces a quorum sensing molecule PQS (pseudomonas quinolone signal) which induces membrane curvature through interactions with lipid A to stabilize and reduce fluidity of the OM. Without PQS, *P. aeruginosa* struggles to reach the membrane curvature necessary to form OMVs. However, this proposed mechanism appears specific to *P. aeruginosa* cells (Mashburn and Whiteley, 2005; Mashburn-Warren et al., 2008).

More recently, Roier et al. (2016) have proposed a new general regulatory model based on the maintenance of lipid asymmetry pathway (MLA) that could be widely applicable to Gram-negative bacteria. The MLA pathway has been shown to play a role in the retrograde trafficking of phospholipids (PLs) from the outer leaflet of the OM to the inner membrane. The core components of this pathway are highly conserved in Gram-negative bacteria (Malinverni and Silhavy, 2009). The pathway consists of an inner membrane ABC (ATP binding cassette) transporter consisting of MlaBDEF (MlaB is absent in α and ε *proteobacteria*), a periplasmic chaperone MlaC, and the OM lipoprotein MlaA. The components of the MLA pathway are homologous to YrbBCDEF and VacJ in *Haemophilus influenzae*. Roier et al. proposed that down-regulation or mutation of the MLA pathway or components of this pathway will lead to an accumulation of PLs in the outer leaflet of the OM. This accumulation leads to an asymmetric expansion which eventually results in the formation of an OMV. Roier et al. demonstrated increased OMV production in *mla* mutants, as well as reduced *mla* gene expression in *H. influenzae* under iron-limiting conditions. The same study suggested that iron limitation signaled entry to the host nasopharynx, and the subsequent increase in OMV production was a potential mechanism to defend against antibody and complement-mediated attack.

A range of virulence factors have been identified within *C. jejuni* OMVs, including the cytolethal distending toxin and putative virulence-associated proteases that contribute to *C. jejuni* invasion into intestinal epithelial cells (Lindmark et al., 2009; Elmi et al., 2016, 2018). *C. jejuni* OMVs isolated at 37°C (human body temperature) have also been shown to differ in protein abundance compared to 42°C (avian body temperature), suggesting *C. jejuni* can alter cargo in response to host signals (Taheri et al., 2019). An important feature of a pathogen is the ability to sense the environment and to appropriately alter and co-ordinate the regulation of virulence factors. We have previously reported the potential significance of the bile salt sodium taurocholate (ST) in the regulation of OMV-mediated virulence in *C. jejuni* (Elmi et al., 2018) and have demonstrated increased OMV production in the presence of physiologically relevant concentrations of ST. In addition, the OMVs isolated from cultures supplemented with ST (ST-OMVs) exhibited increased proteolytic activity, cytotoxicity, and immunogenicity to intestinal epithelial cells. ST has also been shown to up-regulate virulence gene expression in *Vibrio cholerae* by causing alterations in disulphide bond formation in TcpP (transmembrane transcription factor) (Yang et al., 2013).

This study further investigates the link between the MLA pathway and OMV production in Gram-negative bacteria. Increased OMV production without compromising membrane stability was observed in a *C. jejuni* 11168 *mlaA* mutant. This study also builds on previous work linking the bile salt ST to increased OMV production, by identifying a potential mechanism for the ST-OMV phenotype. OMV production was observed to increase in the *C. jejuni* wild-type strain, but not in the *mlaA* mutant in the presence of ST. Growth of *C. jejuni* in the presence of ST also resulted in reduced expression of *mla* genes. The data in this study supports the MLA model of OMV production and the link between bile salts and potential virulence regulation in *C. jejuni*.

## Materials and Methods

### Bacterial Strains and Growth Conditions

The *C. jejuni* wild-type strains used in this study were NCTC 11168 (the widely studied human clinical isolate) and 488 (a recent Brazilian human isolate obtained from Daiani Teixeira Da Silva). Cultures were grown in a microaerobic chamber (Don Whitley Scientific, United Kingdom) containing 85% (v/v) N_2_, 10% (v/v) CO_2_, and 5% (v/v) O_2_ at 37°C either on blood agar (BA) plates containing Columbia agar base (Oxoid, United Kingdom), 7% (v/v) horse blood (TCS Microbiology, United Kingdom), and *Campylobacter* Selective Supplement (Oxoid), or in Brucella broth (Oxoid) shaking at 75 rpm unless otherwise stated. *C. jejuni* strains were grown on BA plates for 24 h prior to experiments unless otherwise stated. *Escherichia coli* XL2-Blue MRF' competent cells (Stratagene, United Kingdom) were used for cloning and were grown at 37°C in aerobic conditions either in Lysogeny broth (LB) (Oxoid) shaking at 200 rpm or on LB agar plates (Oxoid). When required, ampicillin (100 μg/ml), kanamycin (50 μg/ml), apramycin (60 μg/ml), or chloramphenicol (50 μg/ml for *E. coli* or 10 μg/ml for *C. jejuni*) were added to cultures.

### Construction of *C. jejuni* Mutants

The *C. jejuni* 11168 *mlaA* gene was inactivated by deletion of the reading frame by homologous cross-over and replacement with a kanamycin resistance cassette. The mutation vector was created using the Gibson isothermal assembly method as previously described (Taylor et al., 2017). The *C. jejuni* 11168 wild-type strain was transformed by electroporation and mutant clones selected for kanamycin resistance. Correct insertion and orientation of the kanamycin cassette into the genome was confirmed by PCR.

The *C. jejuni* 488 *mlaA* mutant was constructed by natural transformation using genomic DNA from the *C. jejuni* 11168 *mlaA* mutant spotted onto the *C. jejuni* 488 wild-type strain. Transformations were performed on Mueller-Hinton agar (Oxoid) plates for 5 h under microaerobic conditions at 37°C. Transformants were screened on BA plates supplemented with kanamycin, then putative mutants verified by PCR and sequencing.

### Complementation of *C. jejuni mlaA* Mutants

Complementation of the *C. jejuni* 11168 *mlaA* mutant was performed using the pRRA system to generate a complementation vector as previously described (Taylor et al., 2017). The 11168 *mlaA* mutant was transformed with the complementation vector by electroporation and clones selected for apramycin resistance. Correct insertion of the expression cassette into the genome was confirmed by PCR. In the 11168 *mlaA* complement strain, the inserted *mlaA* gene is under the control of the native promoter.

The *C. jejuni* 488 *mlaA* mutant was complemented using the pCJC1 complementation vector as previously described (Gundogdu et al., 2011; Jervis et al., 2015). Briefly, the complete wild-type gene was amplified by PCR to contain the native ribosome binding site, start codon, and stop codon with primers pCJC1mlaA(F/R) (Table 1) to introduce a Nco1 site at the 5′ end and a Nhe1 sit at the 3′ end. The amplified gene was digested with Nhe1 and Nco1 and ligated into the pCJC1 complementation vector which was used to insert the wild-type gene copy into the 488 ortholog of the *C. jejuni* 11168 pseudogene *cj0223*. Putative clones were selected on BA plates supplemented with chloramphenicol then verified by PCR and sequencing. In the 488 *mlaA* complement strain, the inserted *mlaA* gene is under the control of a constitutive chloramphenicol promoter.

**Table 1:** Primers used in this study.

**Primer name**	**Primer sequence 5^**′**^-3^**′**^**	**Source**
GSmlaA(F)	TAGGAGTTTTTGCTGAG	This study
GSmlaA(R)	GCTAAGTTCATTGCGTC	This study
ISAmlaA(F1)	GAGCTCGGTACCCGGGGATCCT CTAGAGTCTGGCACTACAATAAATAAGG	This study
ISAmlaA(R1)	AAGCTGTCAAACATGAGAACCAAGG AGAATGTTTTGGTATTCTTGTTCAA	This study
ISAmlaA(F2)	GAATTGTTTTAGTACCTAGCCAAGGT GTGCATTTATATCCATTCTTGCGT	This study
ISAmlaA(R2)	AGAATACTCAAGCTTGCATGCCTGCAGG TCGCCAGTTGTTATTATCATTG	This study
pCJC1mlaA(F)	CCCCCATGGTTTTAGGAGTTTT TGCTGAGAATAAAATTTATATC	This study
pCJC1mlaA(R)	CCCGCTAGCTTATTTGCTAAGTTCATTG	This study
pRRAmlaA(F)	ACACTCTAGATTTAGGAGTTTTTGCTTG	This study
pRRAmlaA(R)	ACACCAATTGGATTAAAAATATTTT TTTCATTAA	This study
qRTmlaA(F)	GATCCTACTTGGGCAAGTATAGC	This study
qRTmlaA(R)	ATGCTTACGAGCAAAGACGCAATG	This study
qRTmlaC(F)	CCACTTCTATGGTAGTAGATGGG	This study
qRTmlaC(R)	GTAGGGCATCAAAGCCTTGGTT	This study
rpoA(F)	CGAGCTTGCTTTGATGAGTG	Ritz et al., 2009
rpoA(R)	AGTTCCCACAGGAAAACCTA	Ritz et al., 2009

*GS, gene specific; ISA, isothermal assembly; pRRA, C. jejuni complementation vector; pCJC1, C. jejuni complementation vector; qRT, quantitative reverse transcription PCR*.

### 3,3′-dipropylthiadicarbocyanine Iodide (DiSC3) Permeability Assay

DiSC3 is a potentiometric fluorescent probe which accumulates on, and translocates into, hyperpolarized lipid bilayers and is frequently used to measure membrane permeability. Fluorescence was monitored at Ex/Em wavelength of 622/670 nm in a Cary Eclipse (Agilent, United States) fluorescence spectrophotometer. *C. jejuni* cells were washed in 20 mM sodium phosphate buffer, pH 7.4, containing 10 mM KCl, and set to an optical density at 600 nm of 0.1. DiSC3 was added to 5 μM final concentration and fluorescence emission followed for 2 min to establish the baseline. Polymyxin B was then added (100 μM final concentration) and emission followed for a further 1 min, before the addition of SDS (20 mM final concentration) to give the maximum fluorescence value. Percentage incorporation of the dye into the membrane was calculated by:

$$100-\left(\frac{emission\;after\;Polymyxin\;B\left(minus\;baseline\right)}{maximum\;emission\;after\;SDS\;\left(minus\;basline\right)}\right)\times 100$$

### Growth Kinetics and Membrane Sensitivity Assays

The growth of each *C. jejuni* wild-type, mutant and complement strains was characterized by measuring both the OD_600_ value and colony forming units (CFUs) of a liquid culture every 2 h for 18 h. Sensitivity to the bile salt sodium taurocholate at biologically relevant concentrations was analyzed measuring OD_600_ and CFU of liquid cultures supplemented with 0.2% (w/v) ST every 2 h for 18 h. Sensitivity to ST at high concentrations was analyzed by comparing the OD_600_ and CFU of late-log phase broth cultures in the presence or absence of 2% (w/v) sodium taurocholate. Sensitivity to the zwitterionic detergent lauryl sulfobetain (LSB) was analyzed by comparing CFUs of mid-log phase cultures serially diluted and spotted onto Brucella agar with or without supplementation of LSB (1.25 mM final concentration). Sensitivity to polymyxin B was analyzed by comparing CFUs of mid-log phase cultures serially diluted and spotted onto BA plates with and without supplementation with polymyxin B (2.5 μg/ml final concentration).

### Outer Membrane Vesicle Isolation

*C. jejuni* OMVs were isolated as previously described (Elmi et al., 2012). Briefly, *C. jejuni* from a 24 h BA plate were resuspended in Brucella broth and used to inoculate 50 ml of pre-equilibrated Brucella broth to an OD_600_ of 0.1. Cultures were grown to late-log phase, time points determined by growth kinetics for each strain. OD_600_ values were normalized to OD_600_ 1.0 and the sterile supernatants obtained by pelleting cells and filter-sterilizing supernatants through a 0.22 μm membrane (Millipore, United Kingdom). The supernatants were then concentrated to 2 ml using an Ultra-4 Centrifugal Filter Unit with a nominal 10 kDa cut-off (Millipore). The concentrated filtrate was ultra-centrifuged at 150,000 × g for 3 h at 4°C using a TLA 100.4 rotor (Beckman Instruments, United States). The resulting OMV pellet was washed by resuspending in phosphate buffered saline (PBS) and pelleting by ultra-centrifugation as described above. All isolation steps were performed at 4°C and the resulting OMVs pellet was resuspended in PBS and stored at −20°C.

### Quantification of Outer Membrane Vesicles

The protein concentration of OMV preparations was quantified using a commercial bicinchoninic-acid (BCA) assay kit according to the manufactures protocol (Thermo Fisher Scientific, United Kingdom), using BSA as the protein standard. The lipo-oligosaccharide of OMV preparations was quantified by measuring 2-Keto-3-deoxyoctonate acid (KDO) content as described previously (Lee and Tsai, 1999). Briefly, 50 μl samples were hydrolysed with 50 μl sulphuric acid (0.5 N) at 100°C for 15 min, then mixed with 50 μl 0.1 M periodate reagent (H_5_IO_6_) and incubated at room temperature for 10 min. 200 μl 4% (w/v) sodium arsenite reagent (NaAsO_2_) then 800 μl 0.6% (w/v) thiobarbituric acid was added and samples heated again to 100°C for 15 min. Samples were mixed with 1 ml dimethyl sulfoxide (DMSO) to stabilize the chromophore and OD_548_ measurements taken. 2-Keto-3-deoxyoctonate ammonium salt (Sigma-Aldrich, United Kingdom) was used as the KDO standard.

### Analysis of Outer Membrane Vesicles by Nanoparticle Tracking Analysis

Nanoparticle tracking analysis (NTA) was conducted using a ZetaView PMX 110 instrument (Particle Metrix GmbH, Germany) following the manufacturer's instructions. The instrument was calibrated against a known concentration of PS100 nanoparticles with 100 nm diameter (Applied Microspheres B. V., The Netherlands). Nanostandards and OMV samples were suspended in particle-free PBS (Sigma-Aldrich) and diluted appropriately before analysis. Each sample was counted and sized across two cycles of 11 frames per cycle with a flow cell sensitivity of 80%.

### Quantitative Real-Time Polymerase Chain Reaction (qRT-PCR)

To investigate the expression of *mlaA* and *mlaC*, total RNA from both the *C. jejuni* 11168 and 488 wild-type strains, grown in Brucella broth to mid-log phase either in the presence or absence of 0.2% (w/v) sodium taurocholate was extracted using Invitrogen PureLink RNA kit (Invitrogen, United Kingdom). DNA contamination was removed using the TURBO DNA-free kit (Thermo Fisher Scientific). Purified RNA was quantified using a NanoDrop machine (NanoDrop Technologies, United Kingdom) and used to synthesize complementary DNA (cDNA) using the SuperScript III First-Strand Synthesis kit (Thermo Fisher Scientific). qRT-PCR reactions were performed in triplicate using SYBR green master mix (Thermo Fisher Scientific) and an ABI7500 machine (Applied Biosystems). Relative gene expression comparisons were performed using the ΔΔCT (cycle threshold) method (Schmittgen and Livak, 2008) and previously published *C. jejuni rpoA* primers (Ritz et al., 2009) for internal controls to normalize the mean CT of each gene to the stably expressed housekeeping gene *rpoA*.

### Statistical Analysis

At least three biological replicates were performed each in triplicate for each experiment. Statistical analyses were performed using Prism software (GraphPad Software). Variables were compared using a student's *t*-test or a one sample *t*-test with ^*^ indicating a *p* < 0.05, ^**^ indicating a *p* < 0.01, ^***^ indicating a *p* < 0.001, and ^****^ indicating a *p* < 0.0001.

## Results

### Identification of *C. jejuni* Orthologs of the MLA Pathway in *E. coli*

**Figure 1:**
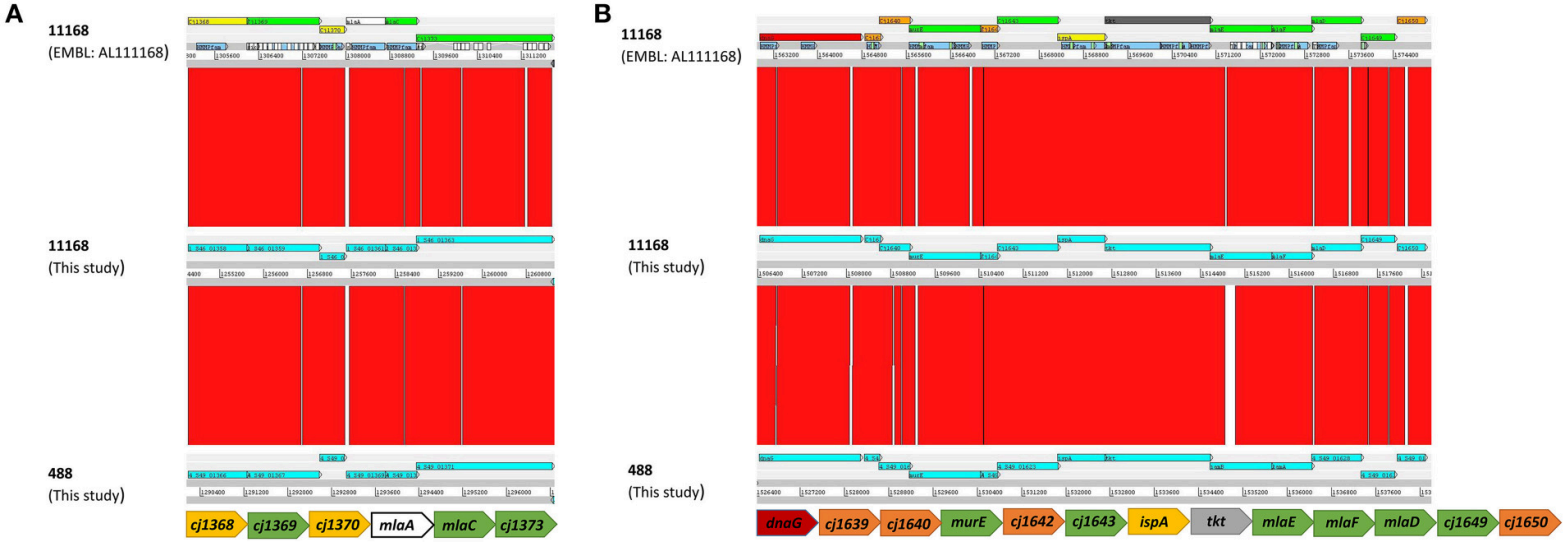
Comparison using the Artemis Comparison Tool (ACT) (Carver et al., 2005) software of the gene clusters containing **(A)** *mlaAC* and **(B)** *mlaEDF* using the *C. jejuni* 11168 original genome sequenced strain from the Sanger public database (ID CJ11168 accession number AL111168) and the new sequences of the 11168 and 488 wild-type strains produced here. Below each ACT window is the conserved organization of the *mla* gene clusters for the strains used in this study based on synteny between the annotated AL111168 genome sequence and the newly sequenced strains in this study. The color qualifier for annotated gene sequences was based on gene function following the scheme adopted by the Wellcome Trust Sanger Institute Pathogen Genomic Department. Functional colors included in this figure are: yellow (central/intermediary/miscellaneous metabolism); dark green (surface–IM, OM, secreted, surface structures); white (pathogenicity/Adaptation/Chaperones); red (information transfer–transcription/translation + DNA/RNA modification); orange (conserved hypothetical), and dark gray (energy metabolism).

The genomes of *C. jejuni* 11168 and 488 contain sets of genes encoding proteins homologous to components of the MLA pathway in *E. coli* and the VacJ and YrbCEFD proteins in *H. influenzae*. The MLA pathway consists of MlaA (outer membrane lipoprotein), MlaC (periplasmic chaperone), and MlaEFD (inner membrane ABC transporter). In *C. jejuni* these proteins are encoded in two separate regions of the genome (Figure 1). Cj1371 and Cj1372 in *C. jejuni* 11168 are within the same gene cluster and are homologous to MlaA and MlaC in *E. coli* (amino acid sequence similarity of 29.4 and 21.6%, respectively, to *E. coli* MG1655 MlaA and MlaC) and VacJ and YrbC in *H. influenzae* (amino acid sequence similarity of 25.8 and 22.2%, respectively, to *H. influenzae* KW20 VacJ and YrbC). Homologous proteins were also identified in *C. jejuni* 488 with 97.4 and 96.8% amino acid similarity to Cj1371 and Cj1372, respectively. Cj1646, Cj1647, and Cj1648 in *C. jejuni* 11168 are encoded by the same gene cluster and are homologous to MlaEFD in *E. coli* (amino acid sequence similarity of 27.6, 31.8, and 18.8%, respectively, to *E. coli* MG1655 MlaEFD), and YrbEFD in *H. influenzae* (amino acid sequence similarity of 27.7, 36.4, and 22.8%, respectively, to *H. influenzae* KW20 YrbEFD). Homologous proteins were also identified in *C. jejuni* 488 with 98.1, 99.2, and 97.6% amino acid similarity to Cj1646, Cj1647, and Cj1648, respectively. *mlaB* or *yrbB* were absent in the *C. jejuni* strains used in this study. ε proteobacteria have previously been reported to lack *mlaB* homologs (Roier et al., 2016). The MLA nomenclature will be used for this study. The *C. jejuni* 11168 published annotated whole genome sequence (Sanger database: ID CJ11168 accession number AL111168) has assigned color qualifiers based on predicted functionality. MlaDEFC are predicted to be functionally linked to cell surfaces (IM, OM, secreted, surface structures). MlaA is predicted to be functionally linked to pathogenicity, adaptation, and chaperones (Figure 1).

### No Change in Sensitivity of *mlaA* Mutant to Detergent Compared to Wild-Type Strains

**Figure 2:**
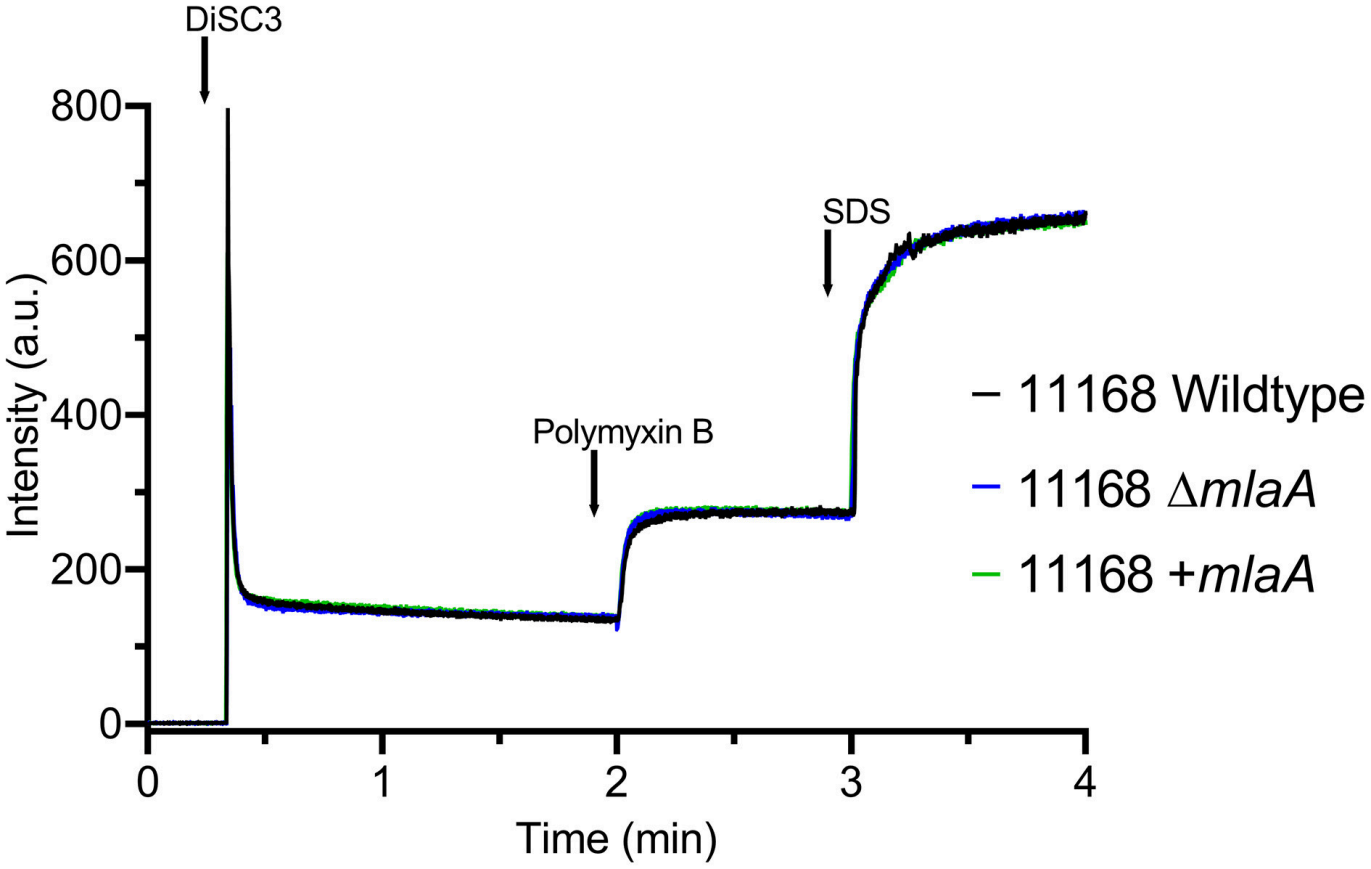
Membrane permeability of the *C. jejuni* 11168 wild-type strain, isogenic *mlaA* deletion mutant (Δ*mlaA*), and complemented (+*mlaA*) strain. Permeability of intact cells was measured using the fluorescent probe 3,3'-Dipropylthiadicarbocyanine Iodide (DiSC3) which accumulates on, and translocates into, hyperpolarized lipid bilayers. Cells were washed prior to the assay, removing OMVs, to measure parent cell membrane permeability. Polymyxin B was added to the cells at a final concentration of 100 μM and release of DiSC3 from the membrane was measured. SDS was added at a final concentration of 20 mM to measure maximum fluorescence.

As the MLA pathway is proposed to play a role in the maintenance of the bacterial OM (Malinverni and Silhavy, 2009), the membrane integrity of the *mlaA* mutants was investigated to ensure any changes in OMV production were not an artifact of a fragile OM. Membrane permeability of the *C. jejuni* 11168 parent cell membranes in the absence of OMVs was analyzed using the DiSC3 assay. *C. jejuni* cells were washed (removing OMVs) then re-suspended in assay buffer. There was no significant difference in percentage incorporation of the dye after membrane stress with 100 μM final concentration polymyxin B between the 11168 wild-type, *mlaA* mutant or complement strain (Figure 2).

Growth or survival during culture conditions in the presence of detergents (LSB and ST) were used to investigate membrane integrity. No significant difference in growth rates measured by either OD_600_ readings or CFUs was observed prior to stationary phase for either the *mlaA* mutant or the complemented mutant when compared to the respective wild-type strain. There was also no significant difference observed for any strain when grown in the presence of 0.2% (w/v) ST (biologically relevant concentration) compared to the same strain cultured in Brucella broth alone prior to stationary phase. The *C. jejuni* 11168 strain was able to grow to higher CFU and OD_600_ values than the *C. jejuni* 488 strain (Figure S1).

**Figure 3:**
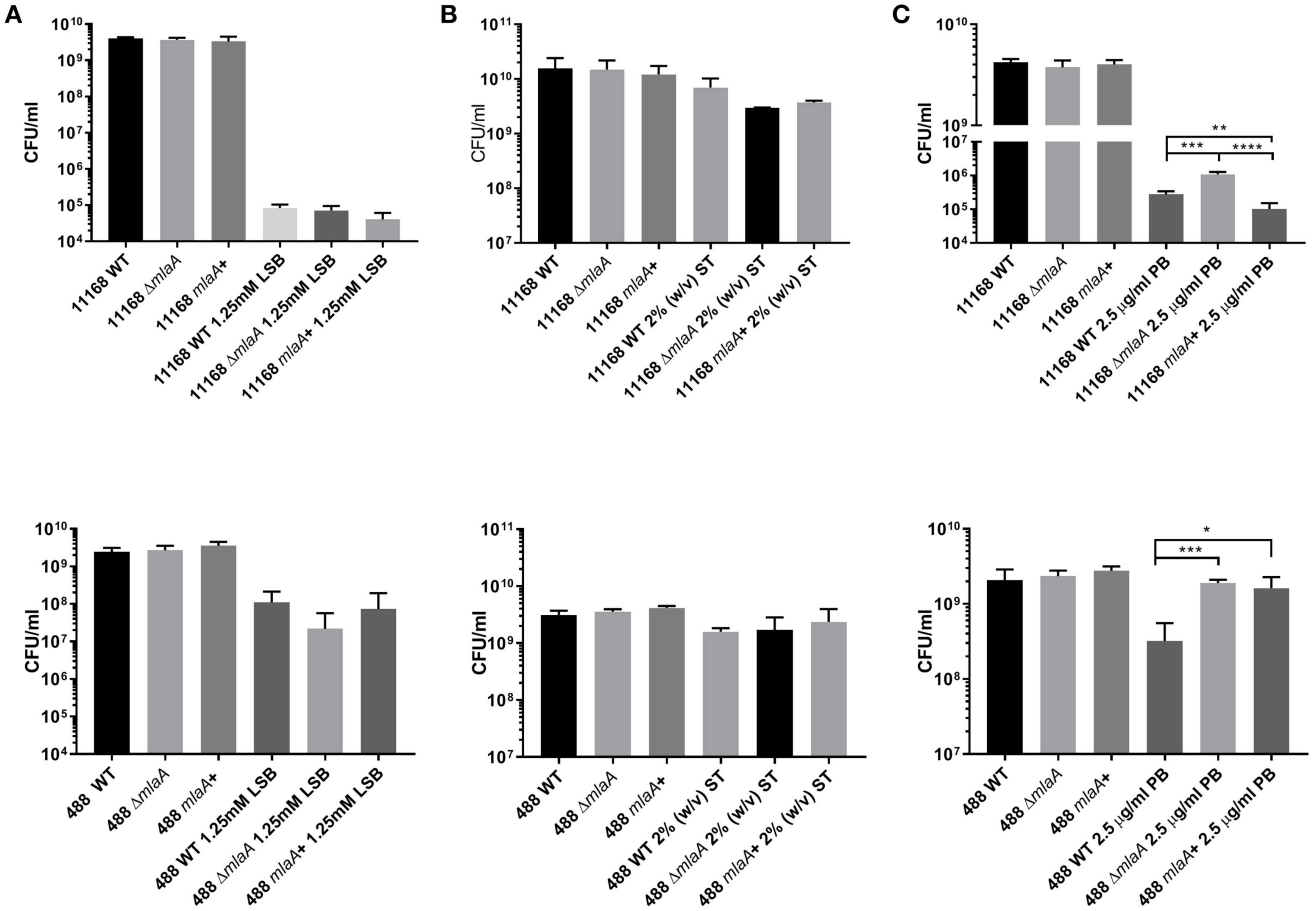
Survival of *C. jejuni* strains exposed to detergent stress or detergent-like stress. **(A)** Mid-log phase cultures spotted onto Brucella agar in the presence or absence of 1.25 mM lauryl sulfobetaine. **(B)** Late-log phase cultures grown in Brucella broth in the presence or absence of 2% (w/v) sodium taurocholate. **(C)** Mid-log phase cultures spotted onto blood agar in the presence or absence of 2.5 μg/ml polymyxin B. ^*^p < 0.05; ^**^p < 0.01; ^***^p < 0.001; ^****^p < 0.0001.

Survival of bacteria in the presence of high concentrations of detergent was investigated either by plating bacteria onto agar supplemented with 1.25 mM LSB or by culturing bacteria in Brucella broth supplemented with 2% (w/v) ST. Survival of both the *mlaA* mutant and complement strains was comparable to the corresponding wild-type strains for both detergents (Figures 3A,B). 1.25 mM LSB caused around a 4-log reduction in growth for 11168 strains compared to around a 2-log reduction for 488 strains. Less than a log reduction in growth was observed for bacteria cultured in 2% (w/v) ST (Figure 3B).

### *mlaA* Mutants Exhibit Increased Resistance to Polymyxin B

Polymyxin B targets cells through binding to LOS or lipopolysaccharide (LPS), meaning OMVs are capable of binding polymyxin B in the extracellular environment (Manning and Kuehn, 2011). Resistance to a low concentration of polymyxin B (2.5 μg/ml) was used as an initial screen for increased OMV production in the *mlaA* mutants compared to the respective wild-type strains. The *C. jejuni* 11168 and 488 *mlaA* mutants were significantly more resistant to polymyxin B compared to their wild-type strains under culture conditions. Complementation of the 11168 *mlaA* mutant with the *mlaA* gene under the control of the native promotor almost completely restored wild-type polymyxin B sensitivity, however, complementation of the 488 *mlaA* mutant with the *mlaA* gene under the control of a constitutive chloramphenicol promotor did not. It would appear that the expression of the inserted *mlaA* gene in the 488 *mlaA* complement is not sufficient to restore the wild-type phenotype under these stress conditions (Figure 3C).

### *mlaA* Mutation Results in Increased OMV Production

**Figure 4:**
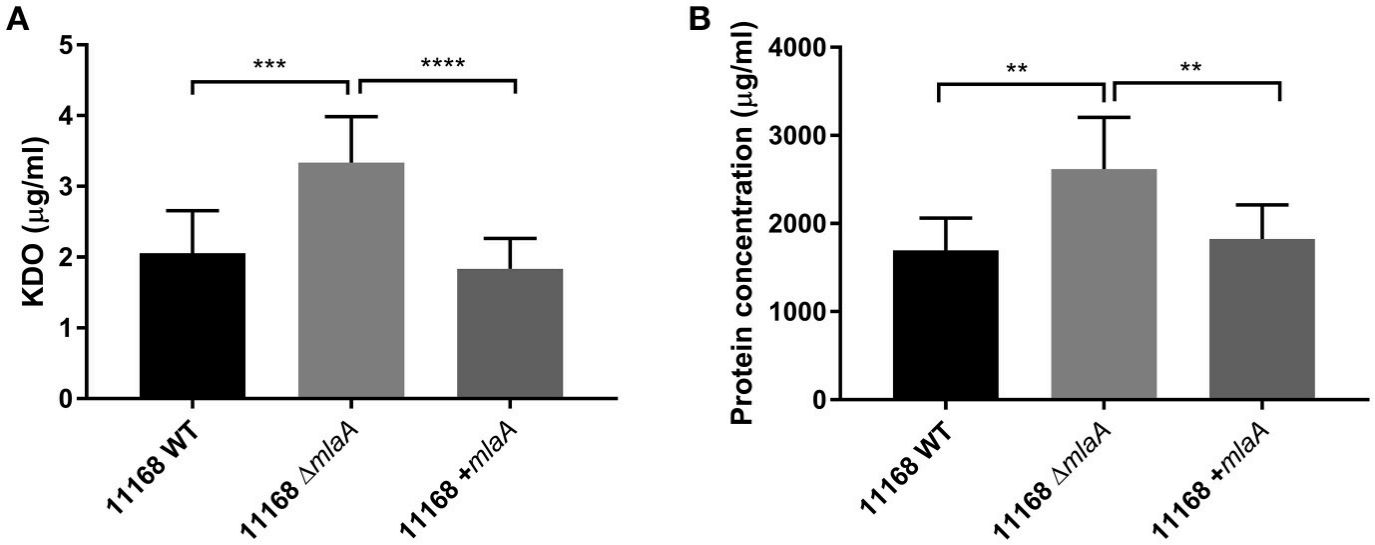
Mutation of *mlaA* increases OMV production. OMVs were isolated from late-log phase *C. jejuni* 11168 cultures grown in Brucella broth. OMV preparations from cultures of *C. jejuni* 11168 wild-type, *mlaA* mutant, and complement strains of equivalent OD_600_ values were quantified by analyzing **(A)** total KDO as a measurement of LOS and **(B)** total protein. ^**^p < 0.01; ^***^p < 0.001; ^****^p < 0.0001.

Total protein and KDO concentration were used to quantify OMV preparations. KDO is a component of LOS and can be used to indicate the amount of OM present. As protein and KDO concentrations are unable to determine if OMV preparations contain more OMVs or larger OMVs, NTA was used to verify any changes in production. OMV preparations were from bacterial cultures of equivalent volume and OD_600_ readings. OMV preparations isolated from the *C. jejuni* 11168 *mlaA* mutant had significantly higher concentrations of both protein and KDO compared to the wild-type and complement strains (Figure 4). The OMV preparations of the *mlaA* mutant strains were also confirmed to contain more OMVs by NTA particle counts (Figure S2).

### Co-culture With Sodium Taurocholate Increases Lipid and Protein Concentration of *C. jejuni* OMVs

**Figure 5:**
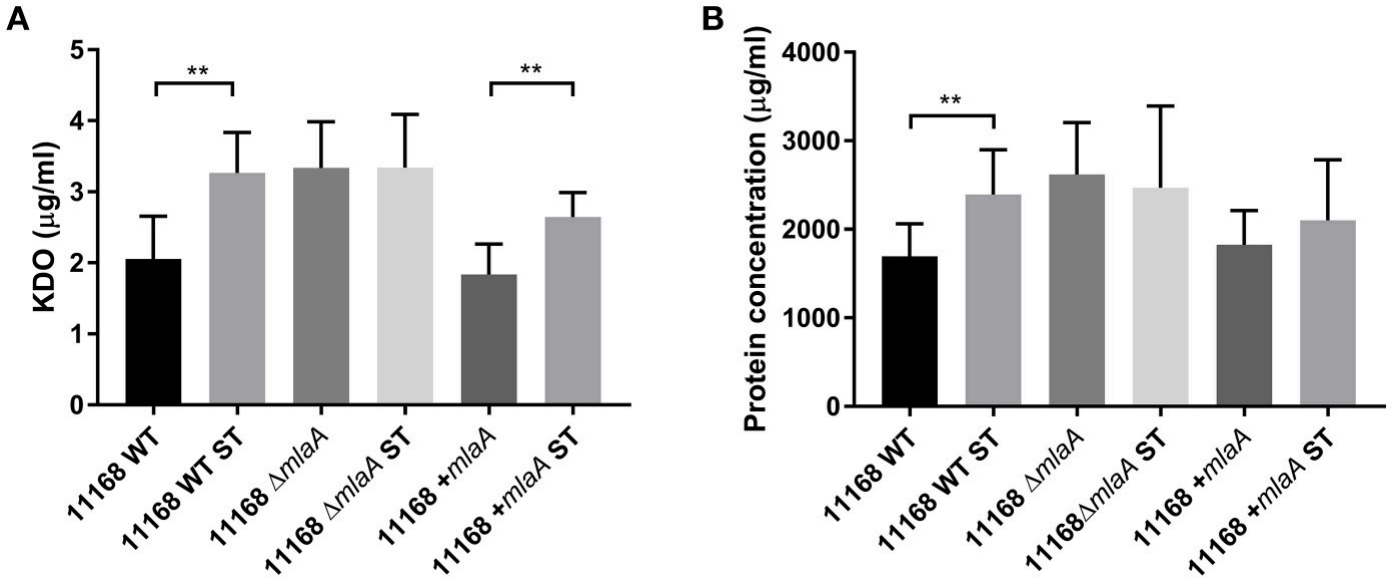
The presence of a biologically relevant concentration of ST increases OMV production in *C. jejuni* wild-type strain but not in the *mlaA* mutant. OMVs were isolated from late-log phase *C. jejuni* cultures grown in Brucella broth either in the presence or absence of 0.2% (w/v) ST. OMV and ST-OMV preparations from cultures of *C. jejuni* wild-type, *mlaA* mutant and complement strains of equivalent OD_600_ values were quantified by analyzing **(A)** total KDO as a measurement of LOS and **(B)** total protein ^**^p < 0.01.

To investigate the link between ST and OMV production proposed by Elmi et al. (2018), OMVs were isolated from *C. jejuni* 11168 wild-type, *mlaA* mutant, and complement strains co-cultured with 0.2% (w/v) ST. OMV preparations isolated from ST supplemented cultures had significantly higher concentrations of KDO for the wild-type and complement strains (Figure 5). ST did not increase the KDO concentration of the *mlaA* mutant OMV preparations. Protein concentrations were significantly higher for the wild-type strain in the presence of ST and higher, but not significantly so for the *mlaA* complement strain. The protein concentration did not increase in the presence of ST for the *mlaA* mutant (Figure 5). The increase in OMVs based on KDO and protein concentrations in the presence of ST for the wild-type and complement strain also correlated to an increase in particle number for NTA (Figure S2).

### Changes in Lipid and Protein Concentration of OMVs Are Inversely Proportional to OMV Size

**Figure 6:**
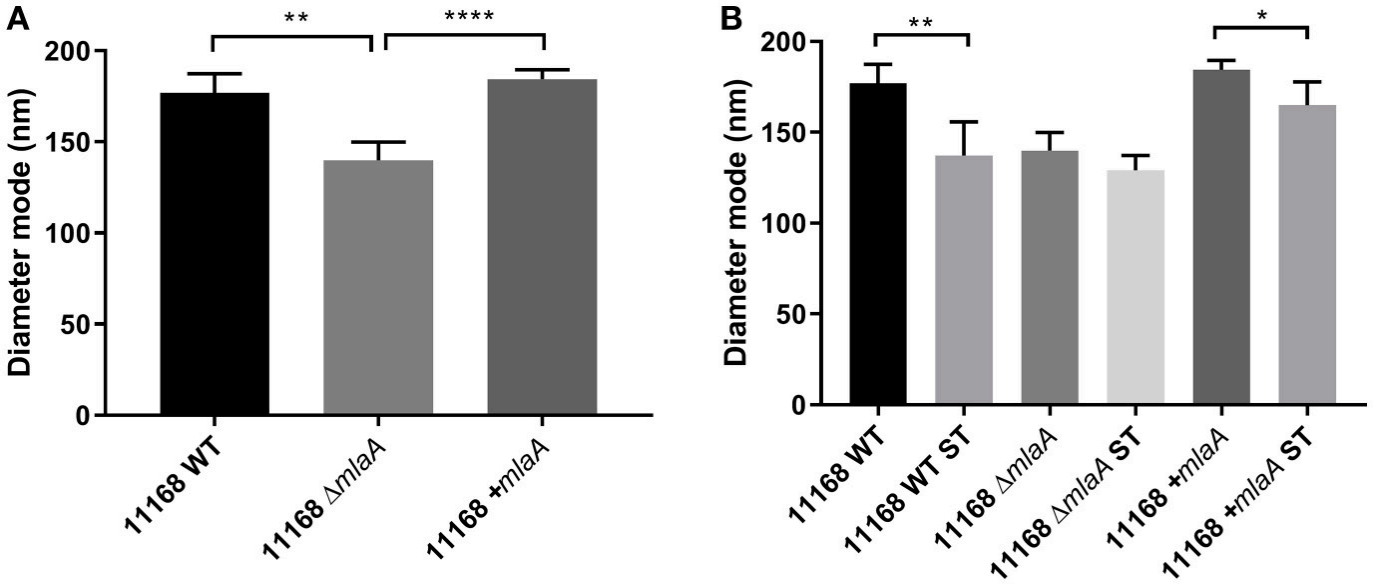
Mutation of *mlaA* or ST exposure increase OMV particle numbers not OMV size. OMVs were isolated from late-log phase *C. jejuni* cultures grown in Brucella broth either in the presence or absence of 0.2% (w/v) ST. OMV preparations from cultures of *C. jejuni* wild-type, *mlaA* mutant, and complement strains of equivalent OD_600_ values were sized by nanoparticle tracking analysis using a PMX 110 ZetaView instrument. **(A)** OMV mode diameter, **(B)** OMV compared to ST-OMV mode diameter. ^*^p < 0.05; ^**^p < 0.01; ^****^p < 0.0001.

In addition to quantifying OMV production levels, the size of the OMVs produced were also investigated by NTA. OMV preparations from all strains and all conditions contained one main size cluster which contained 92–99% of the population. An increase in protein, KDO and particle number either in the presence of ST or in the absence of a complete MLA pathway correlated to a reduction in OMV size. OMVs isolated from the *C. jejuni* 11168 wild-type, *mlaA* mutant, and *mlaA* complement strain had a mode diameter of 177, 140, and 184 nm, respectively, when isolated from cultures grown in Brucella broth. When co-cultured with 0.2% (w/v) ST, ST-OMV mode diameters were 137, 133, and 159 for the 11168 wild-type, *mlaA* mutant, and *mlaA* complement strains, respectively (Figure 6). A similar inverse correlation between OMV quantity and size was also observed for mean OMV diameters (Figure S2).

### Co-culture of *C. jejuni* With Sodium Taurocholate Results in Reduced Gene Expression of *mlaA* and *mlaC*

**Figure 7:**
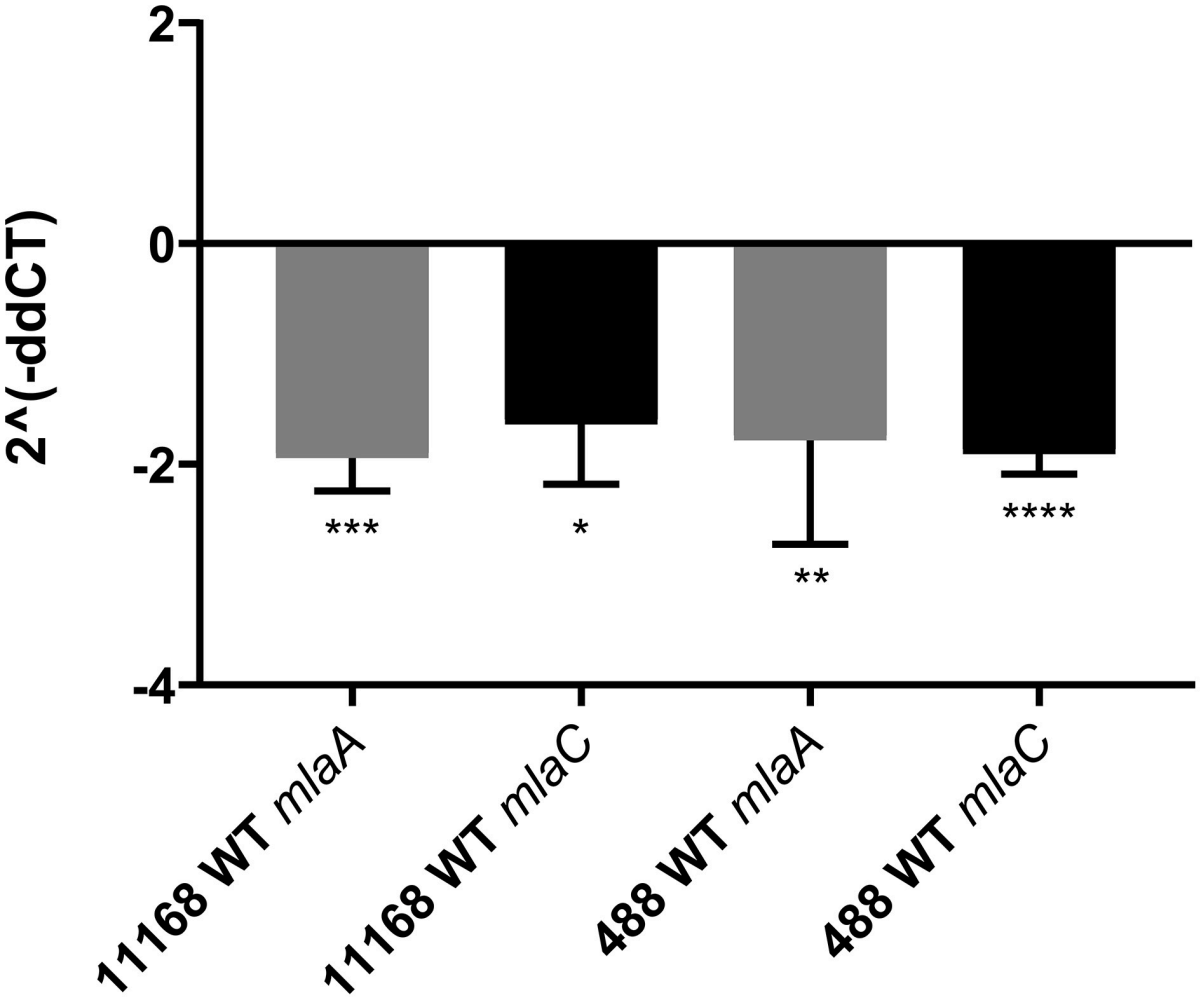
qRT-PCR analysis of *mlaA* and *mlaC* transcription in *C. jejuni* 11168 and 488 wild-type strains co-incubated with 0.2% (w/v) sodium taurocholate. Analysis was performed using *mlaA, mlaC*, and *rpoA* gene specific primers. *rpoA* was used as an internal control. 2^∧^(−*ddCT*) = fold change in gene expression compared to *C. jejuni* grown in the absence of ST. ^*^p < 0.05; ^**^p < 0.01; ^***^p < 0.001; ^****^p < 0.0001.

As the *C. jejuni* 11168 *mlaA* mutant was not observed to increase OMV production in the presence of 0.2% (w/v) ST in contrast to the wild-type strain, the effect of 0.2% (w/v) ST on the relative expression of genes encoding components of the MLA pathway was investigated. *mlaA* and *mlaC* were selected for investigation. RNA was isolated from mid-log phase cultures of the *C. jejuni* 11168 and 488 wild-type strains. A statistically significant approximate 2-fold down-regulation of gene expression of both *mlaA* and *mlaC* in both wild-type strains (11168 and 488) in the presence of ST compared to the absence of ST, was observed relative to the *rpoA* internal control (Figure 7).

## Discussion

Previous characterizations of the cargo of *C. jejuni* OMVs have identified biologically active compounds that contribute to immunogenicity, cytotoxicity, and the breakdown of the gut barrier mediated by bacterial proteases (Jang et al., 2014; Elmi et al., 2016, 2018). The mechanisms regulating OMV production in *C. jejuni* however, are still poorly understood. In this study, we have characterized both the role of the MLA pathway in *C. jejuni* OMV production, as well as the role of ST in regulating the MLA pathway. OMV production in *C. jejuni* was increased either in the presence of a biologically relevant concentration of ST or in the absence of a complete MLA pathway, linking the model proposed by Roier et al. (2016) with the ST phenotype observed by Elmi et al. (2018).

Despite the bacteriostatic effect of bile salts (Begley et al., 2005), in this study *C. jejuni* was shown to be highly tolerant to ST stress, showing less than a log-reduction in growth at 2% (w/v) ST, a concentration higher than is considered to be biologically relevant (Elmi et al., 2018). This then suggests that much lower and biologically relevant concentrations of ST could act as stimuli without compromising bacterial growth or membrane integrity. This is in agreement with a previous study (Elmi et al., 2018), which did not observe any defect in growth of *C. jejuni* associated with a biologically relevant concentration of ST (0.2% w/v). The effect of a combination of bile salts similar to that expected to be found in the human gut was not investigated in this study, however, the resistance to ST demonstrated here highlights the exquisite adaptation of *C. jejuni* to the gut environment where localized signals co-ordinate bacterial behavior.

There is conflicting evidence in the literature regarding the impact of *mla* mutations on membrane integrity. Malinverni and Silhavy (2009) observed increased sensitivity of *E. coli mla* mutants to SDS in combination with EDTA, but not to SDS alone. EDTA is a chelating compound that is thought to destabilize LPS to increase membrane permeability (Finnegan and Percival, 2015). The metabolic cost, rather than the membrane integrity of the *E. coli mla* mutants unable to down-regulate OMV production as well as patching areas effected by EDTA, could be the more likely cause of the increased mutant sensitivity. Abellon-Ruiz et al. (2017) observed growth defects in an *E. coli mlaA* mutant compared to the wild-type strain in the presence of both doxycycline and chlorpromazine. This suggested that the PL accumulation at the OM created transient patches of PL bilayer creating windows of opportunity for small molecules that are readily able to diffuse through PL bilayers to enter the cell. However, as an *mla* mutant is unable to down-regulate the creation of these PL bilayer patches, this membrane defect phenotype would be less applicable to a wild-type strain which would be able to up-regulate OMV production without compromising integrity in favorable conditions and down-regulate production reducing the creation of these patches in unfavorable conditions. Roier et al. (2016) also did not observe any obvious membrane defects for the *mla* mutants in response to detergent stress, supporting the model that increased OMV production is due to a regulated process and is not an artifact of an unstable membrane.

As the membrane of an OMV contains either LOS or LPS, it has been suggested OMVs can confer protection against compounds that target or bind to this component of the bacterial membrane, such as bacteriophage or polymyxin B. This was demonstrated by a study that examined the ability of OMVs to protect against low concentrations of polymyxin B (Manning and Kuehn, 2011). Resistance to this antibiotic is normally achieved through modifications of LOS or LPS preventing the antibiotic binding to the bacterial OM. Manning and Kuehn (2011) observed that only OMVs from polymyxin B sensitive strains conferred protection against polymyxin B when added to a culture of a polymyxin B sensitive bacteria, as polymyxin B was unable to bind to OMVs produced by a polymyxin B resistant strain. Here we demonstrated that there was no significant difference in resistance to polymyxin B between the 11168 wild-type strain and the *mlaA* mutant when cells were washed and supernatant discarded to remove OMVs. The fluorescent probe DiSC3 accumulates on, and translocates into, hyperpolarized lipid bilayers, so the DiSC3 assay was used to investigate changes in bacterial membrane permeability. After the addition of polymyxin B, there was no difference in the change in bacterial membrane permeability of the *mlaA* mutant compared to the 11168 wild-type strain. Based on these results, resistance to a low concentration of polymyxin B (2.5 μg/ml) under culture conditions was investigated as an initial screen for increased OMV production by *mlaA* mutants. A positive correlation between increased OMV production and polymyxin B resistance was observed. As OMV isolation is laborious, this could provide a quick and easy initial screen for OMV production levels.

Both in this study and in a previous study (Elmi et al., 2018), a biologically relevant concentration of ST was shown to stimulate OMV production. 0.2% (w/v) ST was observed to almost double OMV production by the *C. jejuni* 11168 wild-type strain. Mutation of *mlaA* increased OMV production by the same magnitude in the absence of ST, whilst ST did not significantly further stimulate OMV production by the *mlaA* mutant. This increase was also shown by NTA to be due to the presence of increased numbers of OMVs rather than larger OMVs. The increase in OMV production was also associated with a small reduction in OMV size. It is possible that this is due to an increase in PLs in the membrane of the OMVs, based on the model proposed by Roier et al. (2016), altering the membrane curvature during the formation of the OMV. The reduced size of wild-type ST-OMVs was also similar to that of the *mlaA* mutant OMVs. Both the *mlaA* mutant OMVs and wild-type ST-OMVs had comparable diameters. This is in contrast to data from a recent study (Taheri et al., 2018) which did not observe increased OMV production in response to ox bile. Ox bile was used to represent a mixture of bile salts, similar to what would be found in the gut, whereas ST alone was used in this study, a single component of human bile. Bile composition not only varies between species but also between individuals of the same species, as well as depending on the location in the gut (Sjövall, 1959; Falany et al., 1994; Nagana Gowda et al., 2009; Chiang, 2017). For example, in humans the proportion of taurine to glycine conjugated bile salts is diet determined. Humans with a taurine rich diet (animal-based products) will have a higher proportion of taurine conjugated bile salts compared to low taurine diet (Sjövall, 1959; Wojcik et al., 2010; Ridlon et al., 2016). As oxes are herbivores, the bile pool composition maybe low in taurine conjugated bile salts, such as sodium taurocholate. Previous work by Elmi et al. (2018) found the effect of sodium taurocholate on OMV production to be dose-dependent. The low dose of ox bile used in this other study, along with the potentially low proportion of taurine conjugates within the ox bile, could explain the different results observed between these studies.

Roier et al. proposed that iron limitation regulates the MLA pathway in *H. influenzae, V. cholera*, and *E. coli* in a Fur dependent manner. However, a RNA sequencing study did not highlight a link between Fur and the MLA pathway in *C. jejuni* (Butcher et al., 2015). As ST has previously been linked to *C. jejuni* virulence and OMV production, ST was investigated as a potential regulator of the C. *jejuni* MLA pathway. The differential effect ST had on OMV production in the *C. jejuni* 11168 wild-type strain compared to the *mlaA* mutant suggests a direct interaction between ST and the MLA pathway in *C. jejuni*. These are the first data suggesting a role for bile salts in regulating the MLA pathway. The qRT-PCR results in this study showed around a 2-fold down-regulation of *mlaA* and *mlaC* in response to ST exposure, further strengthening the hypothesized interaction between ST and the MLA pathway in *C. jejuni*. As previous studies have suggested removing one component of the MLA pathway prevents functionality (Malinverni and Silhavy, 2009; Roier et al., 2016), this could explain why ST appeared to have no effect on OMV production in a single *mla* mutant.

In summary, the data in this study suggests *C. jejuni* can regulate OMV production in response to host gut signals through changes in expression of the MLA pathway. Increased OMV production without compromising membrane stability was observed in a *mlaA* mutant and a potential mechanism for the ST-OMV phenotype was identified. In the presence of ST, OMV production was shown to increase in the wild-type strain, but not in a *mlaA* mutant. Growth of *C. jejuni* in the presence of ST also resulted in reduced expression of both *mlaA* and *mlaC*. Our data supports the MLA model of OMV production and the link between bile salts and potential virulence regulation in *C. jejuni*. As the gut bile composition is dependent on both diet and the microbiota, this study highlights the potential importance of diet and lifestyle factors on the varying disease presentations associated with gut pathogen infection.

## Data Availability

All datasets generated for this study are included in the manuscript and/or the Supplementary Files.

## Author Contributions

DK and ND conceived this study. CD, AT, AE, JW, JL, AG, and OG all performed experiments that contributed to this manuscript. CD, DK, and ND wrote the manuscript, with significant input from AT, AE, OG, and BW.

### Conflict of Interest Statement

The authors declare that the research was conducted in the absence of any commercial or financial relationships that could be construed as a potential conflict of interest.

## Supplementary Material

Figure S1 Growth curves of *C. jejuni* 11168 wild-type strain compared to the 488 wild-type strain. Strains were grown in Brucella broth under microaerobic conditions at 37°C. Growth was characterized by measuring **(A)** OD_600_ values and **(B)** colony forming units (CFUs) every 2 h for 18 h.

Figure S2 Mutation of *mlaA* or ST exposure increase OMV particle numbers not OMV size. OMVs were isolated from late-log phase *C. jejuni* cultures grown in Brucella broth either in the presence or absence of 0.2% (w/v) ST. OMV preparations from cultures of *C. jejuni* wild-type, *mlaA* mutant, and complement strains of equivalent OD_600_ values were sized by nanoparticle tracking analysis using a PMX 110 ZetaView instrument. **(A)** Particle counts per ml and **(B)** OMV mode diameter, mean and median diameters (nm).
