# Supplementary figures and images for: Sodium Taurocholate Stimulates Campylobacter jejuni Outer Membrane Vesicle Production via Down-Regulation of the Maintenance of Lipid Asymmetry Pathway
Source: Front Cell Infect Microbiol. 2019 May 29;9:177. doi: 10.3389/fcimb.2019.00177 (PMC6549495; doi:10.3389/fcimb.2019.00177)

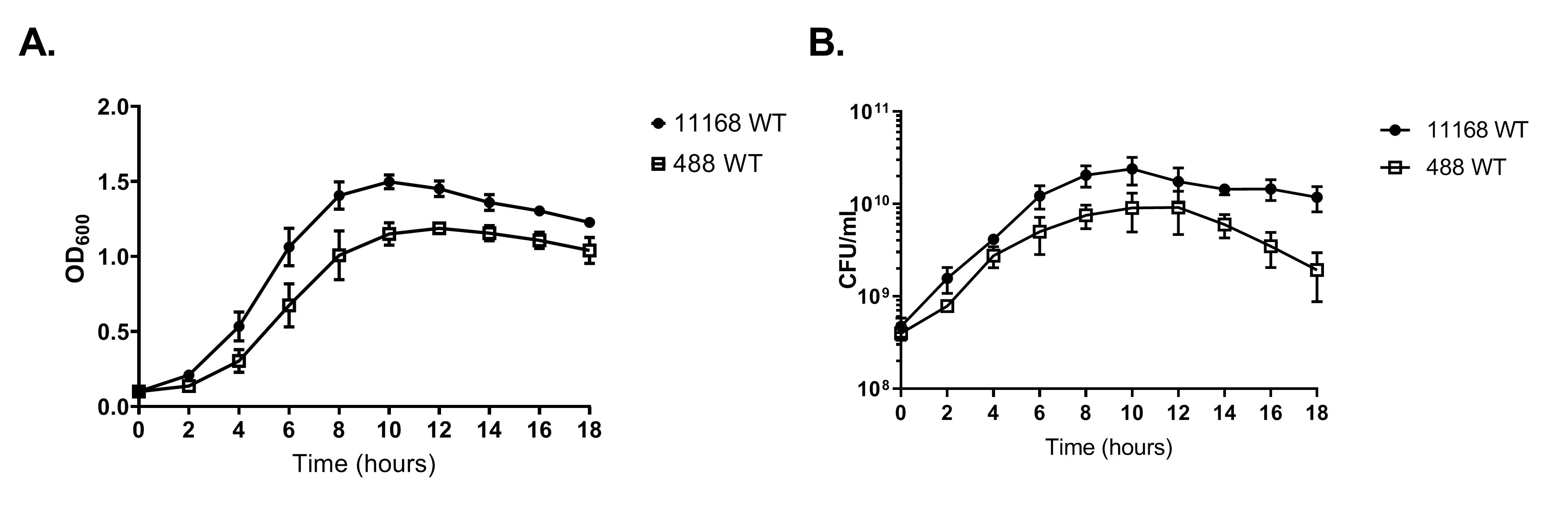

Supplement: Figure S1 — Growth curves of C. jejuni 11168 wild-type strain compared to the 488 wild-type strain. Strains were grown in Brucella broth under microaerobic conditions at 37°C. Growth was characterized by measuring (A) OD600 values and (B) colony forming units (CFUs) every 2 h for 18 h. [file Image_1.TIF]

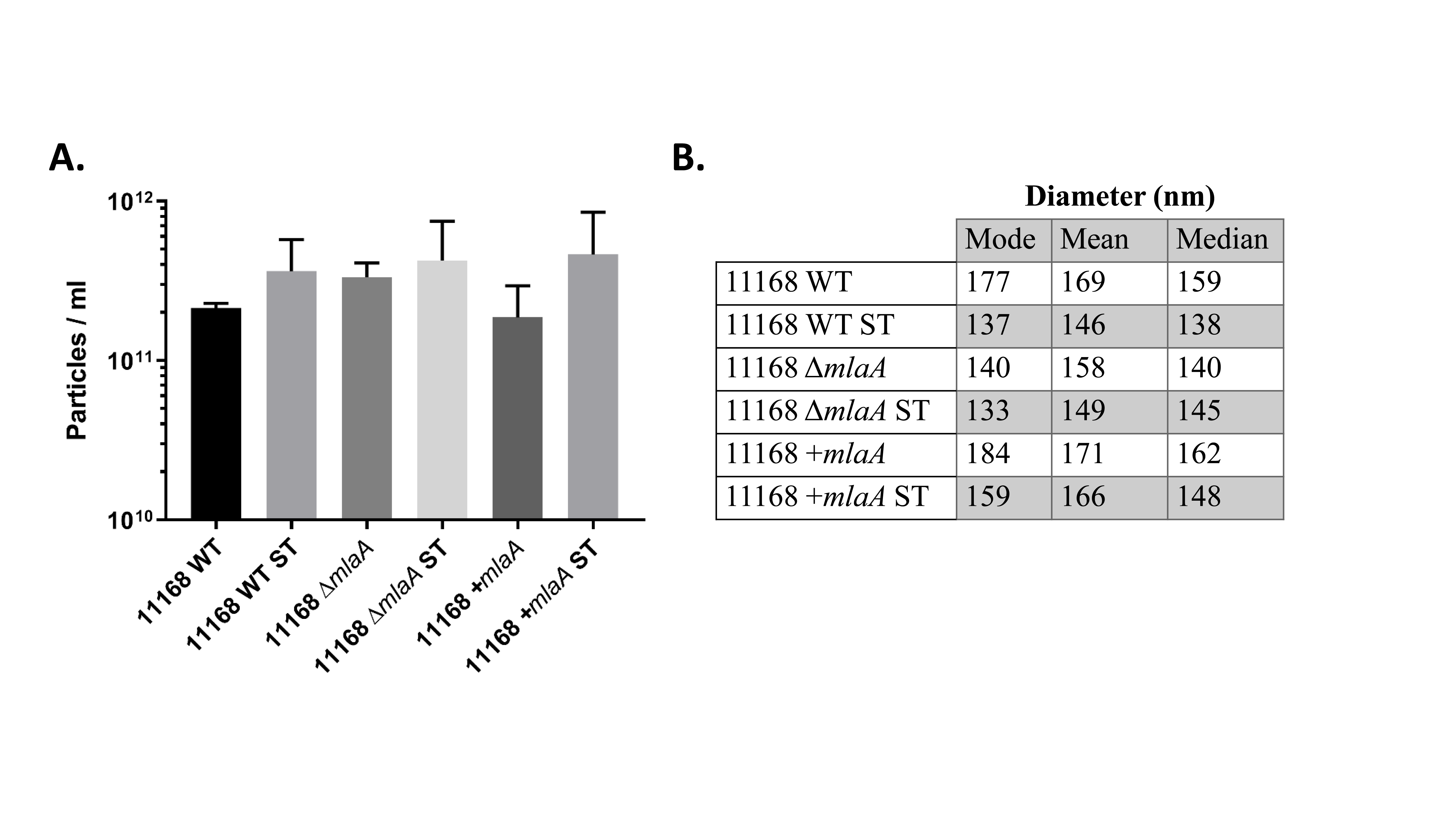

Supplement: Figure S2 — Mutation of mlaA or ST exposure increase OMV particle numbers not OMV size. OMVs were isolated from late-log phase C. jejuni cultures grown in Brucella broth either in the presence or absence of 0.2% (w/v) ST. OMV preparations from cultures of C. jejuni wild-type, mlaA mutant, and complement strains of equivalent OD600 values were sized by nanoparticle tracking analysis using a PMX 110 ZetaView instrument. (A) Particle counts per ml and (B) OMV mode diameter, mean and median diameters (nm). [file Image_2.TIF]
